# PARP1 Stabilizes CTCF Binding and Chromatin Structure To Maintain Epstein-Barr Virus Latency Type

**DOI:** 10.1128/JVI.00755-18

**Published:** 2018-08-29

**Authors:** Lena N. Lupey-Green, Lisa B. Caruso, Jozef Madzo, Kayla A. Martin, Yinfei Tan, Michael Hulse, Italo Tempera

**Affiliations:** a Fels Institute for Cancer Research & Molecular Biology, Lewis Katz School of Medicine at Temple University, Philadelphia, Pennsylvania, USA; b Cancer Biology Program, Fox Chase Cancer Center, Philadelphia, Pennsylvania, USA; University of Southern California

**Keywords:** CTCF, epigenetics, Epstein-Barr virus, latency, PARP1, gene expression

## Abstract

Epstein Barr virus (EBV) is a potentially oncogenic gammaherpesvirus that establishes a chronic, latent infection in memory B cells. The EBV genome persists in infected host cells as a chromatinized episome and is subject to chromatin-mediated regulation. Binding of the host insulator protein CTCF to the EBV genome has an established role in maintaining viral latency type. CTCF is posttranslationally modified by the host enzyme PARP1. PARP1, or poly(ADP-ribose) polymerase 1, catalyzes the transfer of a poly(ADP-ribose) (PAR) moiety from NAD^+^ onto acceptor proteins, including itself, histone proteins, and CTCF. PARylation of CTCF by PARP1 can affect CTCF's insulator activity, DNA binding capacity, and ability to form chromatin loops. Both PARP1 and CTCF have been implicated in the regulation of EBV latency and lytic reactivation. Thus, we predicted that pharmacological inhibition with PARP1 inhibitors would affect EBV latency type through a chromatin-specific mechanism. Here, we show that PARP1 and CTCF colocalize at specific sites throughout the EBV genome and provide evidence to suggest that PARP1 acts to stabilize CTCF binding and maintain the open chromatin landscape at the active Cp promoter during type III latency. Further, PARP1 activity is important in maintaining latency type-specific viral gene expression. The data presented here provide a rationale for the use of PARP inhibitors in the treatment of EBV-associated cancers exhibiting type III latency and ultimately could contribute to an EBV-specific treatment strategy for AIDS-related or posttransplant lymphomas.

**IMPORTANCE** EBV is a human gammaherpesvirus that infects more than 95% of individuals worldwide. Upon infection, EBV circularizes as an episome and establishes a chronic, latent infection in B cells. In doing so, the virus utilizes host cell machinery to regulate and maintain the viral genome. In otherwise healthy individuals, EBV infection is typically nonpathological; however, latent infection is potentially oncogenic and is responsible for 1% of human cancers. During latent infection, EBV expresses specific sets of proteins according to the given latency type, each of which is associated with specific types of cancers. For example, type III latency, in which the virus expresses its full repertoire of latent proteins, is characteristic of AIDS-associated and posttransplant lymphomas associated with EBV infection. Understanding how viral latency type is regulated at the chromatin level may reveal potential targets for EBV-specific pharmacological intervention in EBV-associated cancers.

## Abstract (precis)

EBV is a human gammaherpesvirus that infects more than 95% of individuals worldwide. Upon infection, EBV circularizes as an episome and establishes a chronic, latent infection in B cells. In doing so, the virus utilizes host cell machinery to regulate and maintain the viral genome. In otherwise healthy individuals, EBV infection is typically nonpathological; however, latent infection is potentially oncogenic and is responsible for 1% of human cancers. During latent infection, EBV expresses specific sets of proteins according to the given latency type, each of which is associated with specific types of cancers. For example, type III latency, in which the virus expresses its full repertoire of latent proteins, is characteristic of AIDS-associated and posttransplant lymphomas associated with EBV infection. Understanding how viral latency type is regulated at the chromatin level may reveal potential targets for EBV-specific pharmacological intervention in EBV-associated cancers.

## INTRODUCTION

Epstein-Barr virus (EBV) is a gammaherpesvirus that infects more than 95% of the human population worldwide. While EBV latent infection is typically asymptomatic in healthy individuals, EBV infection is associated with a variety of lymphomas and epithelial cancers and accounts for approximately 1% of all human cancers (1). In particular, EBV-associated lymphomas tend to correlate with specific patterns of viral gene expression or latency type. Understanding how latency type gene expression is regulated may reveal potential therapeutic targets for treating EBV-associated cancers.

During infection of B cells, the EBV genome undergoes a highly regulated process to circularize, chromatinize, repress lytic cycle genes, and selectively transcribe latency genes (2). Accordingly, the virus is subject to chromatin-mediated epigenetic regulation (3, 4). EBV encodes viral proteins and hijacks host proteins to manipulate the chromatin landscape to support the viral life cycle. The host factor CTCF, a protein that functions in maintaining and regulating chromatin architecture, is frequently targeted by DNA viruses to manipulate viral gene expression (5, 6). Indeed, chromatin immunoprecipitation studies have identified a number of CTCF binding sites throughout the EBV genome (7–11). CTCF binding at latency promoters Qp and Cp prevents the spread of repressive chromatin to Qp and mediates latency type-specific promoter usage and gene expression by forming alternative chromatin loops (11, 12). Although CTCF binding is widespread across both the human and EBV genomes, it is unclear how CTCF binding and function are regulated in a site-specific manner.

CTCF is subject to a number of posttranslational modifications that regulate its function, including phosphorylation, SUMOylation, and PARylation (13–15). PARylation is the addition of poly(ADP-ribose) moieties from an NAD^+^ donor onto acceptor proteins, catalyzed by the enzyme PARP1. PARP1 was initially studied in the context of DNA damage, leading to the development and FDA approval of the PARP inhibitor olaparib for the treatment of BRCA-deficient ovarian and metastatic breast cancers (16, 17). More recent work has focused on understanding the mechanisms of PARP1 involvement in transcription and epigenetic regulation (18, 19). PARP1 widely colocalizes with CTCF in human cell lines, and PARylation of CTCF modulates its insulator activity (15, 20, 21). Thus, it is possible that PARylation of CTCF by PARP1 contributes to site-specific regulation of CTCF binding and function.

Previously, our laboratory identified a correlation between EBV latency type and PARP activity (22) and identified a role for PARP1 binding in EBV lytic reactivation (23). Here, we hypothesized that PARP1 cooperates with CTCF to regulate EBV latency. Using genome-wide approaches, we observed widespread CTCF and PARP1 colocalization and identify a cooperative role for PARP1 and CTCF in regulating EBV type III latency at the chromatin level. Taken together, these findings demonstrate that PARP1 regulates EBV latency and provide a rationale for the use of PARP inhibitors in the treatment of cancers associated with type III EBV latency.

## RESULTS

### PARP1 binds specific sites across the EBV genome.

**FIG 1:**
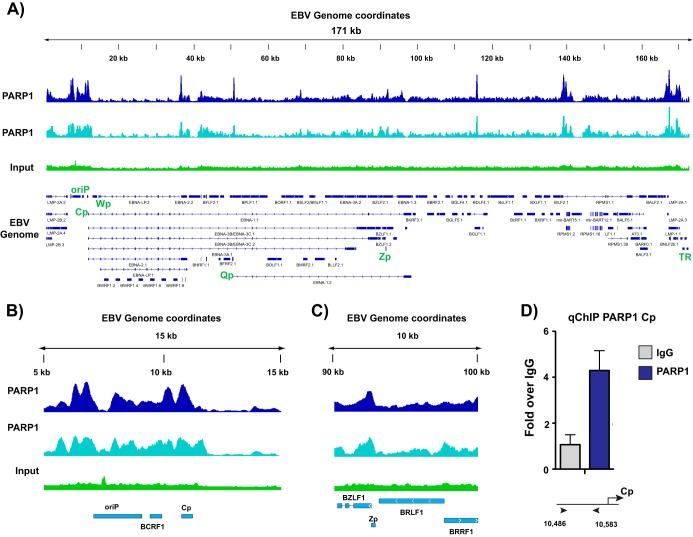
PARP1 binds specific sites across the Epstein-Barr virus genome. (A) Chromatin immunoprecipitation sequencing (ChIP-seq) for PARP1 across the EBV genome in lymphoblastoid cell lines (LCLs) in two biological replicates compared with background input DNA. Peaks are expressed as counts per million reads. Corresponding genes in the linearized EBV genome are shown below. (B) Zoomed image of PARP1 ChIP-seq at the latent Cp locus in LCLs. (C) Zoomed image of PARP1 ChIP-seq at the lytic Zp locus in LCLs. (D) Independent ChIP-qPCR validation of PARP1 enrichment at Cp in LCLs. qPCR data are presented as fold above the level for IgG. Results are representative of three independent experiments and show means ± standard deviations.

Our laboratory previously demonstrated that PARP1 binds to the lytic switch promoter Zp to regulate lytic reactivation (23). Additionally, we have shown that the latency protein LMP1 regulates PARP activity to alter host gene expression and observed a correlation between latency type and levels of PARylation (22). These data prompted us to investigate whether PARP1 activity also regulates viral gene expression, specifically through a DNA binding mechanism, as we observed for reactivation. EBV-immortalized lymphoblastoid cell lines (LCLs) were utilized in these studies, since they exhibit type III viral latency and maintain high levels of PARylation (22). We performed chromatin immunoprecipitation sequencing (ChIP-seq) in LCLs to assess PARP1 binding across the EBV genome (Fig. 1A) and identified numerous PARP1 binding sites that were consistent between biological duplicate experiments. The ChIP-seq experiment validated PARP1 binding at Zp observed previously (Fig. 1C), but most notably it revealed a dense region of PARP1 enrichment around OriP and the active Cp promoter (Fig. 1B). PARP1 binding was independently validated by ChIP-quantitative PCR (qPCR) at the Cp promoter (Fig. 1D).

### PARP1 colocalizes with CTCF across the EBV genome.

**FIG 2:**
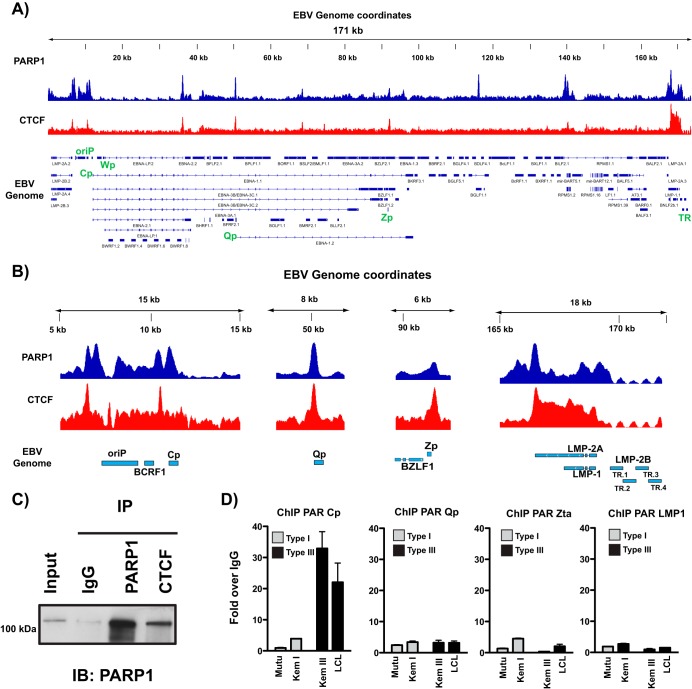
PARP1 colocalizes with CTCF across the Epstein-Barr virus genome. (A) ChIP-seq for PARP1 and CTCF across the EBV genome in LCLs demonstrating widespread colocalization. Peaks are expressed as counts per million reads. Corresponding genes in the linearized EBV genome are shown below. (B) Zoomed images of PARP1 and CTCF ChIP-seq at Cp, Qp, Zp, and LMP1/2 loci. Peaks are shown as counts per million reads. Scale in the *y* axes are independent among the loci shown. (C) Western blot showing PARP1 and CTCF interaction in LCLs. Cell lysates were subjected to immunoprecipitation with antibodies for IgG, PARP1, and CTCF. Immune complexes were resolved by gel electrophoresis and immunoblotted for PARP1. (D) ChIP-qPCR for poly(ADP-ribose) moieties at Cp, Qp, Zp, and LMP1 in representative type I (white bars; Mutu, Kem I) and type III (black bars; Kem III, LCL) latent cell lines. qPCR data are presented as fold above the level for IgG. Results are representative of three independent experiments and show means ± standard deviations.

Work from the Lieberman and Sample laboratories have characterized a prominent CTCF binding site at Cp (7, 8, 12, 24). Based on the established functional relationship between CTCF and PARP1 (20), we also performed ChIP-seq for CTCF in these same LCLs. In comparing PARP1 and CTCF binding across the EBV genome, we identified numerous sites at which PARP1 and CTCF colocalize (Fig. 2A), as well as additional sites in which either PARP1 or CTCF binds alone. PARP1 and CTCF colocalize at both latency promoters, Cp and Qp, the LMP1/2 promoter, and the lytic promoter Zp (Fig. 2B). CTCF and PARP1 can physically interact (25), so we also show by immunoprecipitation that CTCF binds PARP1 in LCL cellular extracts (Fig. 2C). Studies in mice have demonstrated that CTCF can be PARylated by PARP1 (20, 26). To assess whether CTCF is PARylated at these sites of colocalization, we performed ChIP for PAR in a panel of cell lines exhibiting type I and type III latency at the major latent promoters and the lytic promoter Zp (Fig. 2D). Most interestingly, we only observed enrichment for PAR at Cp in type III latent cell lines, suggesting that PARylation of CTCF at this site is important for Cp activation and transcription.

### PARP inhibition alters CTCF binding across the EBV genome.

**FIG 3:**
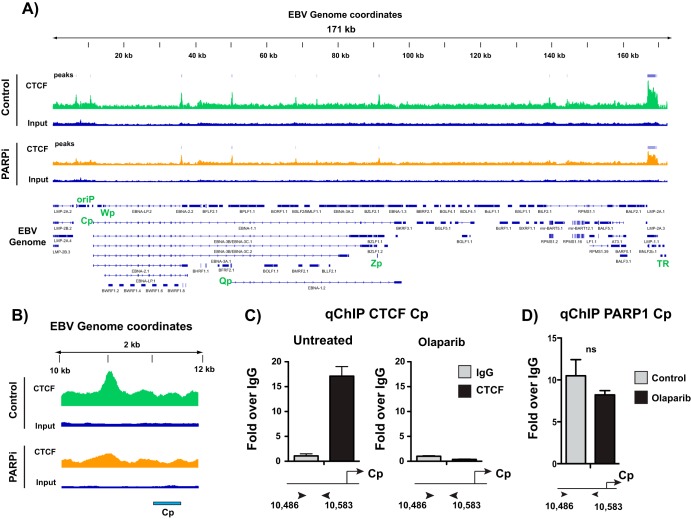
PARP inhibition alters CTCF binding across the Epstein-Barr virus genome. (A) ChIP-seq for CTCF across the EBV genome in untreated or olaparib-treated (PARPi) LCLs and respective input DNA. Peaks are expressed as counts per million reads. Corresponding genes in the linearized EBV genome are shown below. (B) Zoomed image of CTCF ChIP-seq at the latent Cp locus in LCLs, demonstrating the loss of enrichment after olaparib treatment. (C) Independent ChIP-qPCR validation of CTCF enrichment at Cp in untreated or olaparib-treated LCLs. qPCR data are presented as fold above the level for IgG. Results are representative of three independent experiments and show means ± standard deviations. (D) ChIP-qPCR for PARP1 in untreated or olaparib-treated LCLs. qPCR data are presented as fold above the level for IgG. Results are representative of three independent experiments and show means ± standard deviations (ns, not significant).

PARylation of CTCF alters CTCF function (15, 20). Thus, we asked whether the inhibition of PARP activity alters CTCF binding across the EBV genome. Since CTCF is likely PARylated at Cp, we predicted that inhibiting PARP activity would result in a loss of CTCF at Cp. ChIP-seq in LCLs treated with and without the PARP inhibitor olaparib revealed some changes in CTCF binding across the genome (Fig. 3A), although the most prominent change occurred at Cp. CTCF was evicted from the Cp promoter after PARP inhibition (Fig. 3B). By independent ChIP-qPCR, we validated the loss of CTCF binding from Cp with PARP inhibition (Fig. 3C). Because olaparib is known to result in trapping of PARP1 to its DNA targets, we also performed ChIP for PARP1 at Cp in LCLs treated with olaparib. PARP inhibition does not significantly alter PARP1 binding at Cp (Fig. 3D).

### PARP inhibition results in more tightly packed chromatin at Cp.

**FIG 4:**
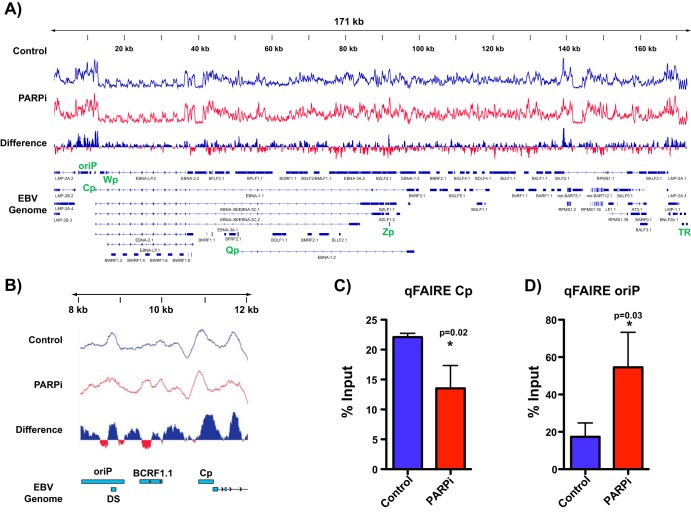
PARP inhibition results in more tightly packed chromatin at the Cp promoter. (A) Formaldehyde-assisted isolation of regulatory element sequencing (FAIRE-seq) to assess chromatin accessibility across the EBV genome in LCLs before (control; blue track) and after inhibition of PARP activity (PARPi; red track). By differential analysis, blue peaks represent regions in which open chromatin is enriched in the control, and red peaks represent regions with more open chromatin in the PARPi sample. (B) Zoomed image of FAIRE-seq at the latent Cp locus in LCLs, demonstrating the packing of chromatin at Cp after PARP inhibition. (C) Independent FAIRE-qPCR validation at Cp and OriP in untreated or olaparib-treated (PARPi) LCLs. qPCR data are presented as percentages of input DNA. Results are representative of three independent experiments and show means ± standard deviations. Statistical significance, as determined by Student's *t* test, is indicated by asterisks and *P* value.

CTCF binding to the genome is integral to maintaining chromatin topology in the mammalian genome (27, 28). The observed loss of CTCF binding after PARP inhibition prompted us to investigate broad changes in chromatin. Accordingly, we used formaldehyde-assisted isolation of regulatory elements (FAIRE), a technique that assays for open and nucleosome-depleted regions of DNA, to detect EBV genome-wide changes in chromatin accessibility in response to PARP inhibition (Fig. 4). We determined chromatin packing in LCLs before (blue track) and after (red track) inhibition of PARP activity. We assessed changes in the FAIRE profile between conditions and determined changes in chromatin accessibility by differential analysis. Blue peaks indicate regions in which more open chromatin is enriched in the control, i.e., chromatin packing after PARP inhibition, while red peaks indicate regions with more open chromatin in the PARP-inhibited sample, i.e., chromatin relaxation after PARP inhibition. Genome-wide the effects on chromatin were varied, resulting in both packing and relaxation of chromatin (Fig. 4A). Upon closer examination of the Cp locus, after PARP inhibition we observed significant chromatin packing at the Cp promoter but, interestingly, chromatin relaxation within OriP (Fig. 4B). FAIRE-qPCR validated the genome-wide observations, showing a loss of open chromatin at Cp with PARP inhibition (Fig. 4C) and enrichment for open chromatin at OriP (Fig. 4D).

### The active chromatin landscape at the Cp promoter is repressed after PARP inhibition.

**FIG 5:**
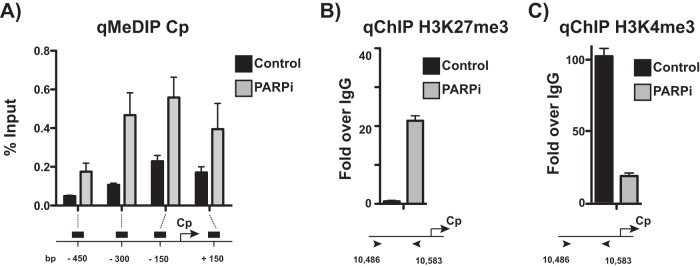
Active chromatin landscape at the Cp promoter is repressed after PARP inhibition. (A) Methylated DNA immunoprecipitation (MeDIP) qPCR at the Cp promoter in untreated and olaparib-treated (PARPi) LCLs. qPCR data are presented as percentages of input DNA. Results are representative of three independent experiments and show means ± standard deviations. (B) ChIP-qPCR for the repressive H3K27me3 mark at Cp in untreated and olaparib-treated (PARPi) LCLs. qPCR data are presented as fold above the level for IgG. Results are representative of three independent experiments and show means ± standard deviations. (C) ChIP-qPCR for the active H3K4me3 mark at Cp in untreated and olaparib-treated (PARPi) LCLs. qPCR data are presented as fold above the level for IgG. Results are representative of three independent experiments and show means ± standard deviations.

In type III latent cells, Cp is associated with active chromatin markers. The chromatin landscape at Cp, at least in part, mediates type III latent gene expression (3, 29). With the observation that PARP inhibition results in packing chromatin at Cp, we further examined the chromatin landscape at Cp. To assess DNA methylation at the Cp promoter, we performed methylated DNA immunoprecipitation to immunoprecipitate methylated DNA from LCLs treated with olaparib or left untreated (Fig. 5A). PARP inhibition increases DNA methylation across the promoter, most significantly just upstream of Cp. Chromatin immunoprecipitation for the repressive histone mark H3K27me3 revealed a gain of enrichment following PARP inhibition (Fig. 5B) and a concomitant loss in enrichment of the active H3K4me3 mark (Fig. 5C), consistent with the packed phenotype observed by FAIRE.

### PARP inhibition alters the EBV latency phenotype, reflecting a transition to type I latency.

**FIG 6:**
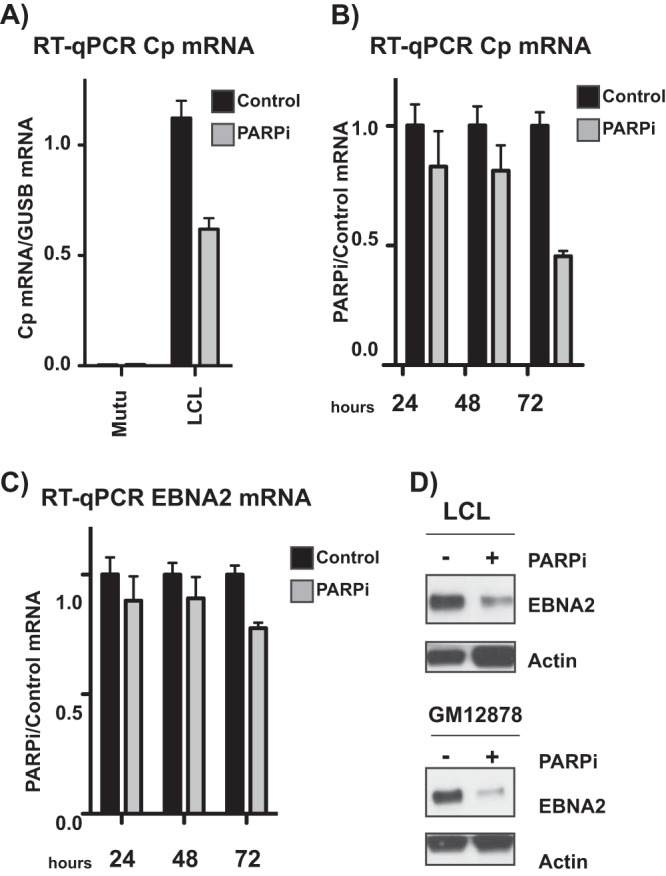
PARP inhibition alters the Epstein-Barr virus latency phenotype, reflecting a transition to type I latency. (A) qRT-PCR showing relative expression of Cp transcripts in untreated and olaparib-treated type I Mutu (negative control) and type III latent LCL cell lines. Expression is relative to that of the abundant B cell transcript *GUSB*. Graph is representative of three independent experiments and shows means ± standard deviations. (B) qRT-PCR showing relative expression of Cp transcripts in LCLs. PARPi cells were treated with olaparib for 24, 48, or 72 h, as indicated. Expression is shown relative to untreated levels. Results are representative of three independent experiments and show means ± standard deviations. (C) qRT-PCR showing relative expression of EBNA2 transcripts in LCLs. PARPi cells were treated with olaparib for 24, 48, or 72 h, as indicated. Expression is shown relative to untreated levels. Results are representative of three independent experiments and show means ± standard deviations. (D) Western blot for EBNA2 protein in two different type III latent lymphoblastoid cell lines treated with olaparib (PARPi). Actin served as a cellular loading control.

PARP inhibition results in the accumulation of repressive chromatin at the active Cp promoter in type III latent cells. Thus, we predicted that treatment with olaparib would decrease transcription from Cp. We performed quantitative reverse transcription-PCR (qRT-PCR) to assess expression from Cp in Mutu and LCL cell lines before and after PARP inhibition, revealing downregulation of Cp (Fig. 6A). Moreover, this downregulation is time dependent over 72 h (Fig. 6B). EBNA2, which is expressed from the Cp promoter, is also downregulated after 72 h of PARP inhibition, although to a lesser extent than Cp itself (Fig. 6C). In addition, at the protein level, EBNA2 expression is significantly decreased to a much greater extent than that at the transcriptional level in two different EBV-immortalized lymphoblastoid cell lines treated with olaparib (Fig. 6D).

### PARP inhibition represses EBV-mediated immortalization and transformation of primary B cells.

**FIG 7:**
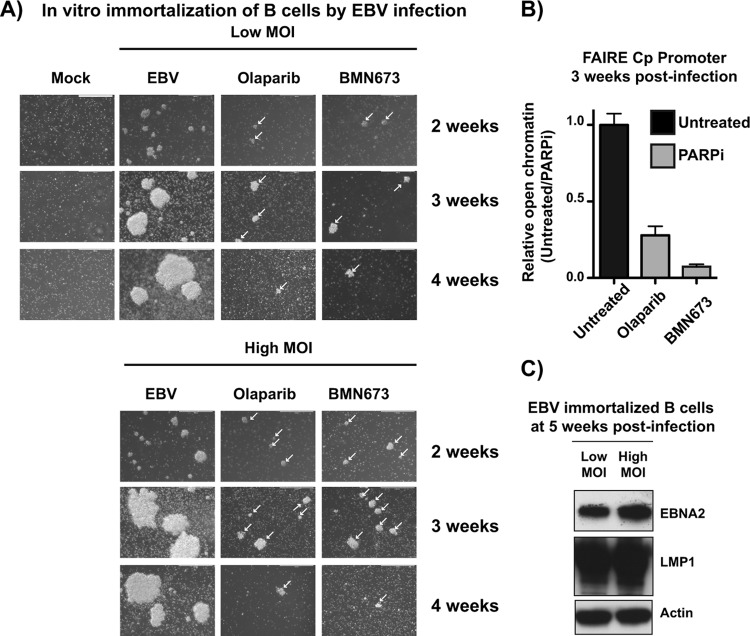
PARP inhibition represses Epstein-Barr virus-mediated immortalization and transformation of primary B cells. (A) Primary peripheral blood mononuclear cells (PBMCs) were treated with the immunosuppressive drug FK506 for 1 h and then incubated with EBV particles at an MOI of 30 (high) or 10 (low). Uninfected PBMCs were used as a control. After 24 h, cells were incubated with the indicated PARP inhibitor. EBV infection was assessed by light microscopy analysis of clusters of EBV^+^ cells over 4 weeks. White arrows indicate clusters in treated samples. (B) Untreated and olaparib- or BMN673-treated (PARPi) cells at 3 weeks were subjected to FAIRE-qPCR to quantify open chromatin at the viral Cp promoter. Results are representative of three independent experiments and show means ± standard deviations. (C) Western blot analysis of EBNA2 and LMP1 expression in untreated samples at 5 weeks postinfection to confirm EBV latency in LCL outgrowths. Actin served as a cellular loading control.

Taken together, these data suggest that PARP inhibition results in the accumulation of repressive marks at Cp, ultimately silencing transcription from Cp and downregulating type III latent protein expression. Cp activation is required for the establishment of EBV latency and transformation of primary B cells. We anticipated that PARP inhibition has a similar effect on Cp during prelatency and may prevent the activation of Cp and establishment of latency, ultimately preventing transformation of primary B cells. Primary peripheral blood mononuclear cells (PBMCs) were incubated with EBV at a low or high multiplicity of infection (MOI) with or without either olaparib or BMN673, a more potent PARP inhibitor, and observed by light microscopy to assess the outgrowth of LCLs (Fig. 7A). Untreated cells began to form clusters by 3 weeks, which were maintained at 4 weeks postinfection. Cellular extracts at 5 weeks were subjected to Western blotting to validate EBV latency; indeed, the cells expressed both EBNA2 and LMP1, reflecting the establishment of type III latency (Fig. 7C). In PBMCs incubated with olaparib or BMN673, clusters were visible at 3 weeks but were significantly smaller than those of the untreated samples (Fig. 7A). By 4 weeks, the PARP-inhibited cells had largely died and were not maintained thereafter. With the cell clusters that had grown out after 3 weeks, we performed FAIRE at Cp to assess chromatin state (Fig. 7B). Consistent with what was observed with PARP inhibition during latency (Fig. 4), the PARP-inhibited samples were significantly less enriched in open chromatin than in the untreated samples, presumably reflecting a transcriptionally nonpermissive environment. Unfortunately, the small number of cells obtained after PARP inhibition was not sufficient to perform additional ChIP and RT-PCR experiments to assess epigenetic marks and transcription status.

## DISCUSSION

Here, we aimed to understand how PARP1 and CTCF cooperate to regulate the EBV genome. We demonstrate that PARP activity has genome-wide effects on CTCF binding across the viral genome and viral gene expression, and that PARylation of CTCF maintains the active chromatin landscape at Cp in type III latency. Taken together, these data elucidate a cooperative role for PARP1 and CTCF in regulating EBV latency and ultimately could provide insight into PARP-based therapeutics for EBV-associated cancers.

**FIG 8:**
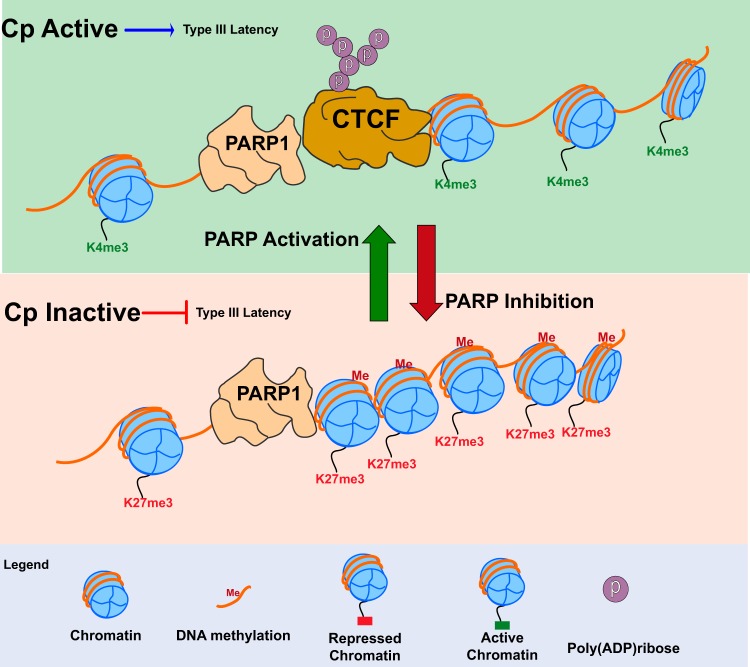
In type III latency, CTCF is PARylated by PARP1 at Cp to maintain the open chromatin landscape and transcription. (Top) During type III latency, PARP1 binds along with PARylated CTCF at the active Cp promoter. PARP1 and CTCF cooperate at Cp to promote open chromatin structure and active transcription by maintaining decondensed nucleosomes and H3K4me3 and preventing DNA methylation. (Bottom) PARP inhibition results in the loss of CTCF at Cp and is associated with the formation of a repressive chromatin environment at the Cp promoter. After PARP inhibition, the Cp promoter becomes enriched for DNA methylation, the repressive H3K27me3 mark, and the nucleosomes are compacted, resulting in decreased type III latency transcription.

We demonstrate that PARP1 and CTCF colocalize at the majority of sites across the EBV genome in LCLs, including at both latency promoters Cp and Qp. However, in type III latent cells, CTCF binding is lost only at Cp after PARP inhibition (Fig. 8). Indeed, in response to PARP inhibition and CTCF loss, Cp undergoes a transition from a transcriptionally active open chromatin landscape to a repressive promoter, gaining both DNA methylation and H3K27me3 marks (Fig. 8). CTCF binding at Qp, however, is maintained. ChIP analysis for PAR suggests that CTCF enriched at Qp is not PARylated and could account for why CTCF enrichment at Qp is not affected by PARP inhibition. Interestingly, ChIP for PAR indicates that in type I latency, CTCF at the active Qp is not PARylated, as might be expected. However, PARP activity is significantly lower in type I latency than type III and may not be relevant for Cp/Qp regulation in type I latency (22). Further, CTCF binding at Qp does not correlate with Qp activity, and stable CTCF knockdown in type I latent cell lines does not alter Qp expression, suggesting that Qp is regulated independently of CTCF/PARP (24, 30).

Although many studies have explored how PARylated CTCF is functionally distinct, no study to date has characterized differentially PARylated CTCF across the genome or how site-specific PARylation might be achieved. It is not fully established how PARP1 is recruited to specific promoters, even in the human genome. PARP1 almost exclusively binds to the EBV genome at CTCF-bound sites. Unlike CTCF, PARP1 does not bind DNA in a sequence-specific manner; it is likely here that PARP1 is recruited to CTCF-bound sites by CTCF or another binding partner. A growing body of work has recently implicated CTCF as having an active role in DNA damage repair. It is intriguing to speculate that CTCF and PARP1 are recruited to the viral genome in a sort of innate immune response related to the double-stranded DNA nature of the viral genome, through which the virus has coopted their roles in transcription for its own advantage.

Of the CTCF sites examined in our work, including those at both Cp and Qp latency promoters, *LMP1* and *BZLF1* promoters, only CTCF at Cp correlated with PAR enrichment by ChIP. In type III latency, Cp is transcriptionally active, while Qp is transcriptionally silenced; it is possible that chromatin marks, lack of DNA methylation, or specific transcription factors associated with an active promoter are required for PARylation of CTCF. In breast cancer cells, PARP1 associates with active chromatin marks, CTCF, and DNase hypersensitivity sites, supporting the idea that CTCF PARylation could preferentially associate with active chromatin marks in the EBV genome (31). PARylation of CTCF as an active chromatin marker would not explain why CTCF at the active *LMP1* promoter is not PARylated in type III latent cells. CTCF binding at LMP1 is unique among other binding sites across the EBV genome for its strong cooccupancy with both cohesin and CTCF (32) and its proximity to a PAX5 binding site (33). It is possible that PARylation regulates CTCF function at some loci (e.g., Cp), while at other sites (e.g., the *LMP1-LMP2* promoter) cohesin or other factors control CTCF. Recently, we showed that LMP1 expression is necessary for the activation of PARP1 observed in type III latently infected cells (22). Thus, a PARylation-independent function of CTCF at the LMP1-LMP2 promoter could represent an evolutionary mechanism to ensure PARP1 activation through LMP1 and subsequent epigenetic regulation during EBV infection.

EBV has likely coopted this mechanism of locus-specific CTCF PARylation for its own advantage. Differential CTCF PARylation at Cp and Qp could dictate CTCF function by latency type. For example, in type III latency, OriP forms a CTCF-mediated chromatin loop with the active Cp promoter (12). It is possible that PARylation of CTCF at Cp in type III latency functions to promote three-dimensional chromatin looping with OriP. As a result, OriP specifically loops with the PARylated Cp promoter but not the unPARylated Qp promoter, maintaining the proper latency type-specific enhancer-promoter interaction. Thus, PARylation of CTCF could serve as a mechanism to stabilize or destabilize alternative chromatin loops across the viral genome in EBV latency. Whether this mechanism is at play during EBV latency remains an open question that is actively under investigation in our laboratory. Nevertheless, our results are consistent with the possibility that the role of PARP1 in EBV latency is dependent on its well-characterized activity in CTCF-mediated chromatin organization and may also involve currently unknown interactions with other epigenetic factors.

PARP1 binding to DNA is mutually exclusive with DNA methylation and tends to associate with CTCF in regions of open chromatin (31). When EBV DNA enters a cell, it becomes gradually methylated, a process that is integral to proper latency establishment and gene expression (34, 35). It is thus possible that the virus has coopted PARP1 and CTCF to stably maintain DNA methylation in the regulation of viral latency. In accordance with our observations in type III latent cells, and because CTCF binding at Cp is important to sustain type III latency, we predicted that PARP inhibition might also prevent the establishment of type III latency. Indeed, we observed that PARP inhibition prevents the outgrowth of LCLs and results in a compact, repressive chromatin landscape at Cp. Due to technical limitations, we were not able to assess viral gene expression in the PARP-inhibited cells, so it is unclear how the few cells that did survive were maintained. We were unable to obtain enough material to perform ChIP to understand how CTCF binding may differ between the untreated and PARP-inhibited cells, although FAIRE indicates that Cp is highly compacted and thus likely repressed at 3 weeks postinfection. Pooling of cells for experiments at earlier time points after infection could provide insight into viral gene expression and chromatin changes and reveal whether viral infection ever occurs in these cells and what might drive the transient outgrowth of the LCLs. Based on our studies in type III latent LCLs, we might predict that CTCF is unable to bind to Cp with PARP inhibition, allowing for the spread of DNA methylation and silencing of Cp. These experiments do not exclude the likely possibility that PARP inhibition also affects the host genome and negatively impacts normal B cell biology to prevent the outgrowth of LCLs from PBMCs. Indeed, work from our laboratory does suggest that PARP inhibition affects transcription of B cell-related pathways (22). However, the less open chromatin conformation structure of the EBV genome in infected cells treated with PARP inhibitors and the repression of viral gene expression in the context of PARP inhibition provide compelling evidence that PARP1 activity is biologically relevant for the EBV epigenome during infection. Although distinguishing the specific role of PARP activity on the host epigenome during viral infection is important, answering this question is beyond the scope of this study. Nevertheless, these data demonstrate an important role for PARP activity in the establishment of EBV latency and outgrowth of immortalized LCLs.

A number of EBV-associated cancers, including posttransplant- and AIDS-associated lymphomas, rely on type III latent protein expression to drive tumorigenesis. Here, we show that PARP inhibition deregulates expression of Cp and subsequently reduces EBNA2 expression. Although EBNA2 is not an oncoprotein *per se*, it promotes several tumorigenic pathways, and its expression is associated with poor survival in diffuse large B cell lymphoma (36–38). Thus, the reduction of EBNA2 expression by olaparib could be an effective therapeutic approach to treat EBV type III latency-associated cancers. We did not, however, observe downregulation of the LMP1 oncoprotein (data not shown). Other work from our laboratory, however, demonstrates that PARP inhibition does attenuate LMP1-induced tumorigenesis in a colony formation assay (22). It is possible that 72 h of PARP inhibition is sufficient to downregulate EBNA2 expression but not long enough to reduce EBNA2 transactivation of LMP1 and ultimately downregulate expression from the *LMP1-LMP2* promoter. Long-term exposure to PARP inhibitors could downregulate LMP1 expression to eliminate expression of the potent oncoprotein. Further, our data suggest that PARP inhibition prevents the establishment of latency, so PARP inhibitors could also help to eliminate viral reservoirs in patients.

In summary, our results, together with previous work from our group and others, reveal a new function for PARP1 activity in the regulation of EBV latency, demonstrating that PARP1 and PARylation are important mechanisms by which the viral epigenome is regulated. Our work adds an important branch to the existing model of how, in the context of EBV latency, PARP1 activity regulates viral chromatin and how two epigenetic factors, CTCF and PARP1, communicate to regulate viral gene expression through modifying chromatin. We acknowledge that this work does not explain at the mechanistic level how PARylation affects the CTCF-mediated three-dimensional organization of EBV chromosome, and we are actively working to characterize the global three-dimensional organization of the EBV genome and further assess the mechanism by which PARP1 alters viral three-dimensional genome structure through CTCF. Gaining a better insight into the PARP1/CTCF interaction may reveal new important molecular steps that are necessary for EBV to regulate viral gene expression during latency.

## MATERIALS AND METHODS

### Cell culture and treatment.

Cell lines were maintained in a humidified atmosphere containing 5% CO_2_ at 37°C. B cell lines were cultured in suspension in RPMI 1640 supplemented with fetal bovine serum at a concentration of either 10% (Mutu and Kem I) or 15% (LCL, Kem III, and GM12878). All cell media were supplemented with 1% penicillin-streptomycin. Olaparib (Selleck Chemical) was dissolved in dimethyl sulfoxide (DMSO) and diluted in the appropriate cell medium for treatment. B cells were treated with 5 μM olaparib for 72 h or as specified. The concentration of olaparib used in experiments does not produce a cellular phenotype and does not induce DNA damage.

### ChIP-seq.

Chromatin immunoprecipitation with next-generation sequencing (ChIP-seq) was performed as previously described (39, 40), with minor changes. A total of 2.5 × 10^7^ cells were cross-linked with 1% formaldehyde for 10 min, and chromatin was sonicated with a Qsonica sonicator. One-tenth of the sonicated chromatin was collected and used as input material, while the rest of the chromatin was immunoprecipitated using 5 μg of CTCF antibody (Millipore), 5 μg of PARP1 antibody (Active Motif), or 5 μg of normal rabbit IgG (Santa Cruz) in LCLs. DNA fragments of ∼150 to 300 bp were visualized by agarose gel purification. Immunoprecipitated DNA was ligated to adapter primers using the TruSeq ChIP library preparation kit (Illumina) and then sequenced using the Illumina HiSeq 2500 platform according to the manufacturer's recommendations (Illumina) at the Fox Chase Cancer Center Sequencing Facility. For both untreated and olaparib-treated LCLs, ∼15 ng of PARP1-immunoprecipitated or input DNA was recovered from each biological replicate, while ∼15 ng of CTCF-immunoprecipitated DNA was recovered after pooling two biological replicates.

Sequencing data for ChIP-seq and input experiments from untreated and treated LCLs were aligned to the EBV genome (GenBank accession number NC_007605) using the Bowtie algorithm (41, 42), and all of the redundant tags were removed before downstream analysis. Peak calling was performed using the Homer algorithm (43) with input DNA used as a reference set. All peaks with false discovery rates (FDRs) of 1% for untreated or treated cells were overlapped to create a list of unique binding sites and used for further analysis. Integrative Genomics Viewer was used for data visualization (44). All ChIP-seq data have been deposited in GEO.

### ChIP-qPCR.

ChIP assays were performed according to the Upstate Biotechnology, Inc., protocol as described previously, with minor modifications (22). Briefly, cells were fixed in 1% formaldehyde for 15 min, and DNA was sonicated using a Qsonica sonicator to generate 200- to 500-bp fragments. Chromatin was immunoprecipitated with polyclonal antibodies to PARP1 (Active Motif), CTCF (Millipore), H3K27me3 (Active Motif), and H3K4me3 (Active Motif). Immunoprecipitation of PAR was carried out using PAR resin (Tulip Biolabs). Real-time PCR was performed with a master mix containing 1× Maxima SYBR green, 0.25 μM primers, and 1/50 of the ChIP DNA per well. Quantitative PCRs were carried out in triplicate using the ABI StepOnePlus PCR system. Data were analyzed by the ΔΔ*C_T_* method (where *C_T_* is threshold cycle) relative to DNA input and normalized to the IgG control. All primers used for qPCR are listed in Table S1 in the supplemental material.

### Immunoprecipitation.

For each immunoprecipitation reaction, 3 × 10^6^ LCLs were washed in cold phosphate-buffered saline (PBS) and resuspended in 3 ml of RIPA buffer (1% NP-40, 1% DOC, 0.1% SDS, 150 mM NaCl, 10 mM Tris-HCl [pH 7.4]). Cells were Dounce homogenized with 10 strokes using a tight-fitting pestle. To allow the SDS to solubilize, the protein cellular solution was rotated at 4°C, and after 30 min the solution was centrifuged at 10,000 × *g* for 5 min. Fifty μl of cell solution was saved as control input. One ml of cell lysate per IP was then used and incubated with 2.5 μg of polyclonal antibodies to PARP1 (Active Motif), CTCF (Millipore), or normal rabbit IgG at 4°C. After overnight incubation, the immunocomplexes were collected by incubating with 100 μl of 50% slurry protein A Sepharose beads (Thermo Fisher Scientific). After 2 h the beads were collected by centrifugation and washed three times with 1 ml low-salt NET buffer (50 mM Tris-HCl, 150 mM NaCl, 5 mM EDTA, 0.5% NP-40 [pH 7.4]) and three times with 1 ml high-salt NET buffer (50 mM Tris-HCl, 300 mM NaCl, 5 mM EDTA, 0.5% NP-40 [pH 7.4]). After the last wash, the beads were resuspended in 50 μl of 2× Laemmli buffer and boiled for 15 min. Immunoprecipitated proteins were resolved by SDS-PAGE gel and analyzed by Western blotting with monoclonal antibody to PARP1 (Trevigen) diluted in 5% PBS plus Tween (PBS-T) according to the manufacturer's instructions.

### FAIRE.

Formaldehyde-assisted isolation of regulatory elements (FAIRE) assay was performed according to an established protocol (45). Per sample, 1 × 10^7^ LCLs were cross-linked with 1% formaldehyde, and the reaction was quenched by adding glycine to a final concentration of 125 mM after 5 min. Cells were washed twice with PBS and cell pellets collected by centrifugation at 300 × *g* for 5 min at 4°C. Fixed cells were then resuspended in cold lysis buffer (1% SDS, 10 mM EDTA, 50 mM Tris [pH 8.0]), and chromatin was sonicated as described for ChIP to generate 300- to 500-bp fragments. One-tenth of the chromatin was saved and used as input material, and the remaining chromatin was diluted with 3 volumes of dilution buffer (0.01% SDS, 1.1% Triton X-100, 1.2 mM EDTA, 16.7 mM Tris [pH 8.0], 167 mM NaCl). Chromatin was centrifuged at 15,000 × *g* for 5 min at 4°C to pellet cell debris, and DNA was isolated from the supernatant by phenol-chloroform extraction and ethanol precipitation. The DNA pellet was resuspended in 100 μl of 10 mM Tris-HCl (pH. 7.5) and incubated with 10 μg of RNase A (New England BioLabs) at 37°C. After 30 min, 20 μg of proteinase K (Roche) was added to the samples and incubated at 55°C for 1 h and then at 65°C to reverse any DNA-DNA cross-links. The de-cross-linked DNA was purified with Zymo Spin I columns and eluted twice with 25 μl of 10 mM Tris-HCl (pH. 7.5). For quantitative PCR analysis, the recovery of FAIRE-extracted DNA was assessed by qPCR and compared to results for input DNA using the ΔΔ*C_T_* method. All primers used for qPCR are listed in Table S1 in the supplemental material.

For next-generation sequencing, 20 ng of input DNA and 20 ng of FAIRE-DNA, each combined from two biological replicates, were used to prepare sequencing libraries using Illumina protocols and subjected to Illumina HiSeq 2500 sequencing according to the manufacturer's recommendations (Illumina). Library preparation and DNA sequencing were performed at the Fox Chase Cancer Center Sequencing Facility. FAIRE-seq analysis was performed as described above for ChIP-seq. All FAIRE-seq data have been deposited in GEO.

### Methylated DNA immunoprecipitation (MeDIP).

DNA was extracted from 2 × 10^6^ cells with a GeneJET genomic DNA purification kit (Thermo Scientific) according to the manufacturer's instructions. A total of 2 μg of DNA was sonicated to between 200 and 300 bp. Samples were boiled for 10 min and immediately cooled on ice. To each sample, 50 μl of 10× immunoprecipitation buffer (IP buffer; 1.4 M NaCl, 0.5% Triton X-100) and 5 μg of 5-methylcytosine antibody (Active Motif) were added, followed by incubation overnight at 4°C. The immunocomplexes were precipitated by adding 50 μl of Dynabeads (Life Technologies) and rotated for 2 h at room temperature. Beads were collected with a magnetic rack and washed three times with 500 μl of 1× IP buffer. Beads were incubated at 50°C for 2 h with shaking in 500 μl of proteinase K digestion buffer (50 mM Tris [pH 8.0], 10 mM EDTA, 0.5% SDS, 1 mg of proteinase K/ml). DNA was extracted twice by phenol-chloroform, followed by ethanol precipitation. DNA was analyzed by quantitative PCR. Two percent of the genomic DNA was used as input material. All primers used for qPCR are listed in Table S1.

### qRT-PCR.

For quantitative reverse transcription-PCR (qRT-PCR), RNA was extracted from 2 × 10^6^ cells using TRIzol (Thermo Fisher Scientific) according to the manufacturer's instructions. SuperScript II reverse transcriptase (Invitrogen) was used to generate randomly primed cDNA from 1 μg of total RNA. A 50-ng cDNA sample was analyzed in triplicate by quantitative PCR using the ABI StepOnePlus system. Data were analyzed by the ΔΔ*C_T_* method relative to expression of the B cell marker GusB and normalized to untreated controls. All primers used for qPCR are listed in Table S1.

### Western blot analysis.

Cell lysates were prepared in radioimmunoprecipitation assay (RIPA) lysis buffer (50 mM Tris-HCl, pH 7.4, 150 mM NaCl, 0.25% deoxycholic acid, 1% NP-40, 1 mM EDTA; Millipore) supplemented with 1× protease inhibitor cocktail (Thermo Scientific). Protein extracts were obtained by centrifugation at 3,000 × *g* for 10 min at 4°C. Protein concentration was measured using a bicinchoninic acid (BCA) protein assay (Pierce). Lysates were boiled with 1× Laemmli sample buffer (Bio-Rad) containing 1.25% β-mercaptoethanol (Sigma-Aldrich). Proteins were resolved by gel electrophoresis on a 4 to 20% polyacrylamide gradient Mini-Protean TGX precast gel (Bio-Rad) and transferred to an Immobilon-P membrane (Millipore). Membranes were blocked in 5% milk in PBS-T for 1 h at room temperature and incubated overnight at 4°C with primary antibodies against PARP1 (Active Motif), EBNA2 (Abcam), LMP1 (Abcam), and actin (Sigma-Aldrich).

### *In vitro* immortalization of peripheral blood mononuclear cells.

Primary peripheral blood mononuclear cells (PBMCs) were purchased from the ATCC (ATCC-PCR-800-011). PBMCs were incubated with the immunosuppressive drug FK506 for 1 h and then incubated with EBV particles at either low MOI (10) or high MOI (30). Control PBMCs were not exposed to EBV particles. After 24 h, EBV-infected PBMCs were incubated with or without either 10 μM olaparib or 100 nM BMN673 to inhibit PARP activity. EBV infection was assessed by evaluating clusters of EBV-positive cells by light microscopy over 4 weeks.

### Statistical analysis.

All experiments presented were conducted at least in triplicate to ensure reproducibility of results. The Prism statistical software package (GraphPad) was used to identify statistically significant differences between experimental conditions and control samples, using Student's *t* test as indicated in the figure legends.

### Accession number(s).

The ChIP-seq and FAIRE-seq data have been deposited in the GEO database under accession number GSE115829.

## Supplementary Material

Supplemental file 1
