# Supplementary material for: PARP1 Stabilizes CTCF Binding and Chromatin Structure To Maintain Epstein-Barr Virus Latency Type
Source: J Virol. 2018 Aug 29;92(18):e00755-18. doi: 10.1128/JVI.00755-18 (PMC6146685; doi:10.1128/JVI.00755-18)
Supplement: Supplemental file 1 [file zjv018183849s1.pdf]

**Table S1. Primers used in qPCR experiments**

| Oligo | Sequence (5' to 3') | Purpose | Genomic location |
|---|---|---|---|
| Cp CTCF bs F' | CACTCGCCCACTAACCTTAAC | ChIP-qPCR | 10486-10506 |
| Cp CTCF bs R' | GGCCTGTAGTTTCGCATCTT | ChIP-qPCR | 10583-10564 |
| Qp CTCF bs F' | CACCTCCCTGATAATGTCTTCAA | ChIP-qPCR | 50009-50031 |
| Qp CTCF bs R' | ACCAGACAACATTACTGTGGAA | ChIP-qPCR | 50085-50064 |
| BZLF1 CTCF bs F' | CTGTCATGGACTCTAGTGTTGTG | ChIP-qPCR | 91212-91234 |
| BZLF1 CTCF bs R' | AGAAGGAGGAAGCAGCCATA | ChIP-qPCR | 91310-91291 |
| LMP1 CTCF bs F' | TCTAGGAAGAAGGCTAGGAAGAA | ChIP-qPCR | 168414-168436 |
| LMP1 CTCF bs R' | CAACGCAGTCTTAGGTATCTGG | ChIP-qPCR | 168515-168494 |
| Cp TSS-3 F' | GTCCACTCACATATTCCACTCTT | MeDIP-qPCR | 10820-10842 |
| Cp TSS-3 R' | GGCCTAGGGTGCATGTTTA | MeDIP-qPCR | 10894-10912 |
| Cp TSS-2 F' | TCGAGTGCTATCTTTGGAACAG | MeDIP-qPCR | 10986-11007 |
| Cp TSS-2 R' | TGAGCTCTCTTATTGGCTATAATCC | MeDIP-qPCR | 11066-11090 |
| Cp TSS-1 F' | ATTCGCCCACGACTTGAAA | MeDIP-qPCR | 11243-11252 |
| Cp TSS-1 R' | AAATTTGCAGCAGAACACAGAG | MeDIP-qPCR | 11313-11334 |
| Cp TSS+1 F' | CCTCATCGCAGGGTTCTTAC | MeDIP-qPCR | 11380-11399 |
| Cp TSS+1 R' | GGCCCTTAGACTTACGGTTTAG | MeDIP-qPCR | 11473-11494 |
| GusB F' | CGCCCTGCCTATCTGTATTC | qRT-PCR | N/A |
| GusB R' | TCCCCACAGGGAGTGTGTAG | qRT-PCR | N/A |
| Cp F' | CCTCATCGCAGGGTTCTTAC | qRT-PCR | 11380-11399 |
| Cp R' | GCTGTTTCTTCAGTCGGTTTAG | qRT-PCR | 11640-11626 |
| EBNA2 F' | CTTAGCCAGTAACCCAGCAC | qRT-PCR | 36095-36108 |
| EBNA2 R' | CGGGTGCTTAGAAGGTTGTT | qRT-PCR | 36185-36166 |
